# The Middle-Aged Man

**Published:** 1921-01-22

**Authors:** 


THE PUBLIC WELL-BEING—(*continued*).

### "The Middle-Aged Man."

At the College of Ambulance, Queen Anne Street, Sir James Cantlie has opened what is lightheartedly described in the hopeful press as "a new crusade against old age." In giving his blessing to this scheme, which appears to comprise a series of exercises suitable for middle-aged persons of sedentary habits, Sir James maintained that the man of forty or fifty should be at his best. As a rule he is not, because he feels, when he becomes middle-aged, that he must give up the activities of his youth. He makes the fatal mistake, it seems, of sinking into an easy chair and taking little or no exercise, with the result that he usually develops rheumatism and dies years before there is the slightest necessity to die. The preventives of old age prescribed by Sir

**THE PUBLIC WELL-BEING**—(*continued*).

James Cantlie are so simple that it seems almost incredible that any ordinary man should die before he is eighty at the least.

This is what he had to say to the middle-aged: "Don't think you ought to be dignified; play about with the children. Avoid easy chairs and carpet slippers. Take plenty of exercise of the right kind. Never think you are too old at seventy. Don't give rheumatism—the chief English complaint—a chance to develop. Take down the old warming-pan from the wall, and use it to dry the bed. Dry your pyjamas and underclothing every day before you put them on." We might add to Sir James Cantlie's modest list another item, and that would be: When you are forty or fifty, don't imagine you are twenty or thirty. In the brittle period of middle age it is almost as easy to bring about disaster by attempting too much as by attempting too little. Not a few middle-aged men who served in the Army, and were absolutely fit to do their own military job, began, after many years of abstinence, to play Association and even Rugby football, and in many cases they were entirely incapacitated in consequence. It is a very good thing to urge men of all ages to take methodical exercise, diet themselves rationally, and to take other elementary precautions to keep themselves in good health. But there is rather an ill-considered tendency at the present time to suggest that there exists some magic formula by which a man need never be so old as he is. Physical fitness is a relative condition, having a somewhat different significance at different ages. We know of no arbitrary device, including the rapturously advertised thyroid gland, which will give back youth to old age. "Putting back the clock" is a pleasant fiction; and the experiences of men famous in the ring or in other branches of sport and athletics who have sought to "come back" have not been encouraging. Middle-age is too often old age, and youth is sometimes middle age; but middle age cannot be youth.

